# Early evidence of royal purple dyed textile from Timna Valley (Israel)

**DOI:** 10.1371/journal.pone.0245897

**Published:** 2021-01-28

**Authors:** Naama Sukenik, David Iluz, Zohar Amar, Alexander Varvak, Orit Shamir, Erez Ben-Yosef

**Affiliations:** 1 National Treasures Department, Israel Antiquities Authority, Jerusalem, Israel; 2 The Mina and Everard Goodman Faculty of Life Sciences, Bar Ilan University, Ramat-Gan, Israel; 3 Environmental Sciences and Agriculture Department, Beit Berl College, Beit Berl, Israel; 4 The Martin (Szusz) Department of Land of Israel Studies and Archaeology, Bar Ilan University, Ramat-Gan, Israel; 5 Department of Archaeology and Ancient Near Eastern Cultures, Tel Aviv University, Tel-Aviv, Israel; Universita degli Studi di Milano, ITALY

## Abstract

In the context of a broad study aimed at examining dyeing technologies in the Timna textiles collection, three samples of prestigious fibers dyed with murex sea snail were identified. Our identification is based on the presence of *6-monobromoindigotin* and *6*,*6-dibromoindigotin* components (detected using HPLC analysis), which is considered unequivocal evidence for the use of murex-derived purple dyestuff. Furthermore, by comparing the analytical results with those obtained in a series of controlled dyeing experiments we were able to shed more light on the specific species used in the dyeing process and glean insights into the ancient dyeing technology. The samples originated from excavations at the extensive Iron Age copper smelting site of “Slaves’ Hill” (Site 34), which is tightly dated by radiocarbon to the late 11^th^–early 10^th^ centuries BCE. While evidence for the important role of purple dyes in the ancient Mediterranean goes back to the Middle Bronze Age (early 2^nd^ millennium BCE), finds of dyed textiles are extremely rare, and those from Timna are the oldest currently known in the Southern Levant. In conjunction with other observations of the very high quality of the Timna textiles, this provides an exceptional opportunity to address questions related to social stratification and organization of the nomadic society operating the mines (early Edom), the “fashion” of elite in the region during the early Iron Age, trade connections, technological capabilities, and more.

## Introduction

### Early iron age textiles from Timna Valley

Textile dyeing has been practiced since prehistoric times, using dyes that are extracted from both plant and animal sources. The color of textiles provides a window into various aspects of ancient societies, including the role of textile dyeing and technological achievements, fashion, social stratification, agriculture and trade connections [1,2]. However, textiles are rare in the archaeological record. Like any perishable organic material, they are usually subject to rapid decomposition and their preservation requires special conditions to prevent destruction by microorganisms [1,3]. Such conditions exist in the ancient copper-ore district of the Timna Valley (southern Israel). Starting in 2013, excavations in several copper production sites in the region by the Central Timna Valley Project [4,5] uncovered dozens of fragments of dyed textiles, which currently constitute the largest Iron Age assemblage in the Southern Levant [6]. This rare assemblage from Timna provides a window into aspects of past societies that are usually hardly visible in the archaeological research. Focusing on the dyeing technologies, we applied High Pressure Liquid Chromatography (HPLC) analyses to identify organic dyestuff; we found three items that were dyed with true purple, which is based on extracts from the murex sea snail. True purple—also known as ‘royal purple’—was considered the most prestigious dye for textiles in many ancient societies. The finds from Timna currently constitute the earliest physical evidence on dyed fiber of true purple in the Southern Levant. Their context—in a marginal region inhabited by semi-nomadic tribes, and within debris of copper smelting activities—sheds new light on the society that operated the mines at the turn of the 1^st^ millennium BCE, with implications for our understanding of trade and economy in the broader Southern Levantine region during this period.

### True purple in antiquity

**Fig 1:**
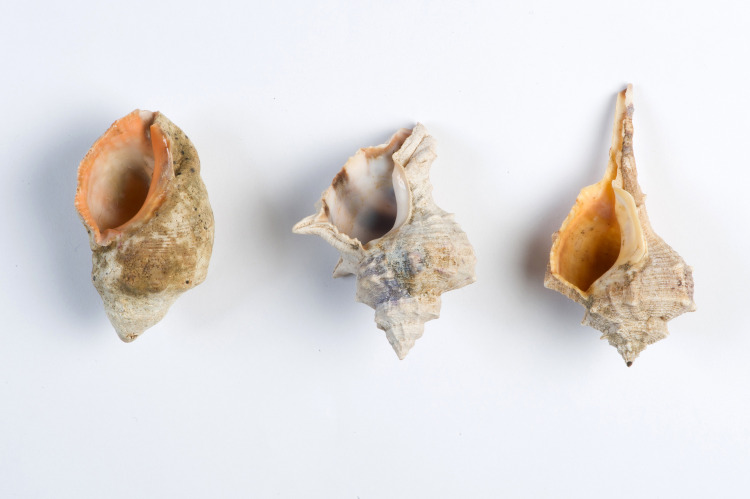
Shells of the three species of murex snails. left to right: *S*. *haemastoma*, *H*. *trunculus* and *B*. *brandaris* (Photo by Shahar Cohen).

True purple was produced primarily from three species of sea mollusks of the Muricidae family, which were common in the Mediterranean Sea: *Hexaplex trunculus* (*Murex trunculus*), *Bolinus brandaris* (*Murex brandaris*) and *Stramonita haemastoma* (*Thais haemastoma*) (Fig 1). This is in contrast to the “imitation purple” dye that was manufactured using various techniques that were based on much cheaper materials than the dyes from the sea snail [7,8]. True purple does not fade easily (as described by the Roman Period author Plutarch [9]). Together with the high complexity of the dyeing process, the small quantity of dyestuff that each snail contains (0.9 g on average in *H*. *trunculus*, for example [10]), it is easy to understand how purple textiles turned into an object of desire and the color itself into a symbol of nobility [11–13]. The true purple colors range in shades from purplish-red to violet-blue. Dyeing is based on material extracted from the snail’s hypobranchial gland (located under the mollusc’s mantle [14]), and the exact shade depends on different parameters, such as the chemical precursors compounds of each snail species, the dyeing process, and the levels of oxygen and light that the dye is exposed to [15]. The dyeing process using murex was a sophisticated, multi-stage process [15]. It was more complex than dyeing techniques based on plants: The snail-harvest [15,16] and the extraction of the gland from the snail required knowledge of biology and much more efforts than collecting plants in the field [17–19]; murex-based dyeing must take place close to the site from which the snails originate, because the freshness of the material has a significant effect on the results [19–22]; and finally, the dyeing process is long and involves biochemical, enzymatic and photochemical reactions, and requires reduction and oxidation processes that probably took several days [19].

Because the purple dyeing technology is no longer practiced [10], our knowledge is based on textual sources like the description of Pliny the Elder (Plinius, NH: IX; see also [19,23]), and inferences from traditional indigo dyeing, which although simpler, involves similar processes (such as the ones related to redox and alkalinity conditions) and is still practiced today in different parts of the world [18,24]. Experimental dyeing also provides insights on the ancient dyeing technology. Although most experiments use strong chemical reagents to shorten the process to a few minutes (like in the hydrosulfite vat method, see S1 Appendix), successful dyeing has been achieved also in experiments based on materials that were available in antiquity [17–19,23,25]. The murex dye belongs to the ‘vat dye’ group that is not soluble in water, thus it requires an alkaline environment. This was achieved by adding various materials such as natural natron, or wood ash of alkaline plants [19,26,27], urine, or even broken shells (S1 Appendix). Reconstructing the reduction stage, which in modern vats is achieved by sodium hydrosulfite and requires non-aerobic conditions, is much more complicated [17]. A number of options have been raised, including the material of the vessel itself (tin or lead, [27]), honey, or madder roots with bran (known as indigo reduction agents [28]), but all failed in experiments. The only successful reconstruction is based on the bacteria in the flesh of the snail itself [17–19]. In this reconstruction, the dye-solution was heated at a moderate temperature (around 50°C) for a few days, until it turned yellow, indicating a *leuco* (soluble) form, and that reduction was completed. In the last stage, after about three days, the fleece or textile was dipped in the reduced dye-solution and then left to oxidize in the air. Once oxidation took place, the dye turned purple.

It is not entirely clear when murex was first used for dyeing, bur the first archaeological evidence that regards the purple dyeing industry are indirect evidence which includes heaps of murex shells, that are dated to around the 19^th^ century BCE. In most cases, this early evidence is associated with the Minoans [29–32]. However, recent archaeomalacological analyses raised the possibility of a multiregional origin of the purple dyeing industry, including regions outside the Aegean [33]. Direct evidence to the use of shellfish purple was identified in the islands of Santorini and Rhodes, where purple pigments from murex snail were found in wall paintings dated to the 17th century BCE [34,35]. The earliest textual evidence for the use of true purple in the Near East is attribute to the murex shells according to textual context is from Akkadian tablets from Nuzi, Mesopotamia, dating to 1425 BCE (this identification is based on contextual and philological arguments [36,37]). Other early written sources are the Amarna letters dated to the 14^th^ century BCE, in which items made of wool dyed with the prestigious purple are described among other merchandise [38]. According to some scholars, the Phoenicians received their name because of their important role in the dyeing industry (assuming the original meaning of ϕoῖνιξ was “red-purple”, [32,39]). The first use of the term ‘royal purple’ is found in a clay tablet from Knossos in Crete dated to the 13th century BCE [40,41]. In the Hebrew Bible, the words *argaman* and *tekhelet* appear side by side and most probably represent a purple hue and a violet-blue hue respectively (similar to the Akkadian words *argamannu* and *takiltu* [42]). It is widely accepted that both colors were based on murex snails, while *tekhelet* is specifically linked to *Hexaplex trunculus* by most scholars [43–45] (although it should be noted that the specific hue is still enigmatic [45]) Both hues are mentioned in the Hebrew Bible in relation to clothing of people of the highest social rank (for example, the garments of the High Priest, Exodus 28:6 [46], and the Midianite kings, Judges 8:26) and religious activities (for example, activities at the tabernacle, Exodus 35:35). Wool dyed with *argaman* and *tekhelet* was mentioned in Sennacherib’s Cylinder among other expensive materials like talents of gold and silver that Hezekiah king of Judah gave him from his palace and the temple in Jerusalem [47]. Most of the textual evidence relating to royal purple comes from the Classical era. It also indicates that purple textiles were highly valued and served as a symbol of prestige, social status and power. Among the various sources are Pliny the Elder [48], Aristo [22] and Vitruvius [49].

In contrast to the many sources that mention purple textiles and the dyeing industry, archaeological evidence is still extremely limited. Common evidence of the purple-dye industry in archaeological sites are the heaps of murex shells, from which the dye-glands were removed [25,50–52]. Such heaps, however, may be evidence of other uses of the snails, including food, lime productions, ceramic temper material, decoration and more [14,33,53,54]. Their association with the dyeing industry is typically based on archaeological indicators such as industrial facilities, working surfaces, indicative tools and alike [55]. Large quantities of crushed shells with a hole in the area of the hypobranchial gland also suggest a deliberate cracking activity as part of the dyeing industry [53]. Still, in some cases crushed murex-shell deposits are difficult to interpret [33]. Some researchers propose a recycling hypothesis, according to which the waste from the purple-dye industry was used for other purposes [33,55].

Important direct evidence of the purple industry can be found in stains of purple on pot sherds. The dye remains are found in most cases on the upper part of ceramic basins, on the inside surface, the areas in which the reduced dye-solution was exposed to air, and underwent oxidation that turned it purple [19,56]. Pottery sherds stained with true purple were found in Sarepta in Lebanon— 14^th^–13^th^ centuries BCE [57], Ugarit (Minet el-Beidha)— 15^th^–13^th^ centuries BCE [52], Tel Keisan— 11^th^ century BCE [19], Tel Shikmona— 10^th^–9^th^ centuries BCE [58] and Tel Kabri— 7^th^ century BCE [19]. These finds indicate the important role of coastal sites in this industry.

Only a small number of textiles dyed with true purple were found in excavations, and until this study, the few that were found in Israel were all dated to the Roman period: two textiles from Masada [59,60] and three from a cave in Wadi Murabba‘at, in the Judean Desert [61]. It should be emphasized that the rarity of true purple textiles predominantly reflects the limited preservation of textiles in the archaeological record; nevertheless, within the aforementioned assemblages (Masada, Murabba‘at and now Timna) true purple textiles are the rarest.

Similar picture also emerges from research in other regions of the ancient Old World, which yielded only scant remains of pre-Roman textiles dyed with shellfish-based dyes. The earliest true purple textiles were found in Syria and are dated to the early second millennium BCE. These include finds from a burial at Chagar Bazar (18^th^–16^th^ centuries BCE, [15]), and samples preserved in gypsum found in Qatna’s palace [62]. The latter were found together with other precious artefacts, including gold beads and jewelry, and were associated with the royal elite [62]. Early evidence of true purple textiles also includes samples from a burial in Stamna (Aetolia, Western Greece) dated to the 12^th^–11^th^ centuries BCE [63].

### The archaeological finds and their context

**Fig 2:**
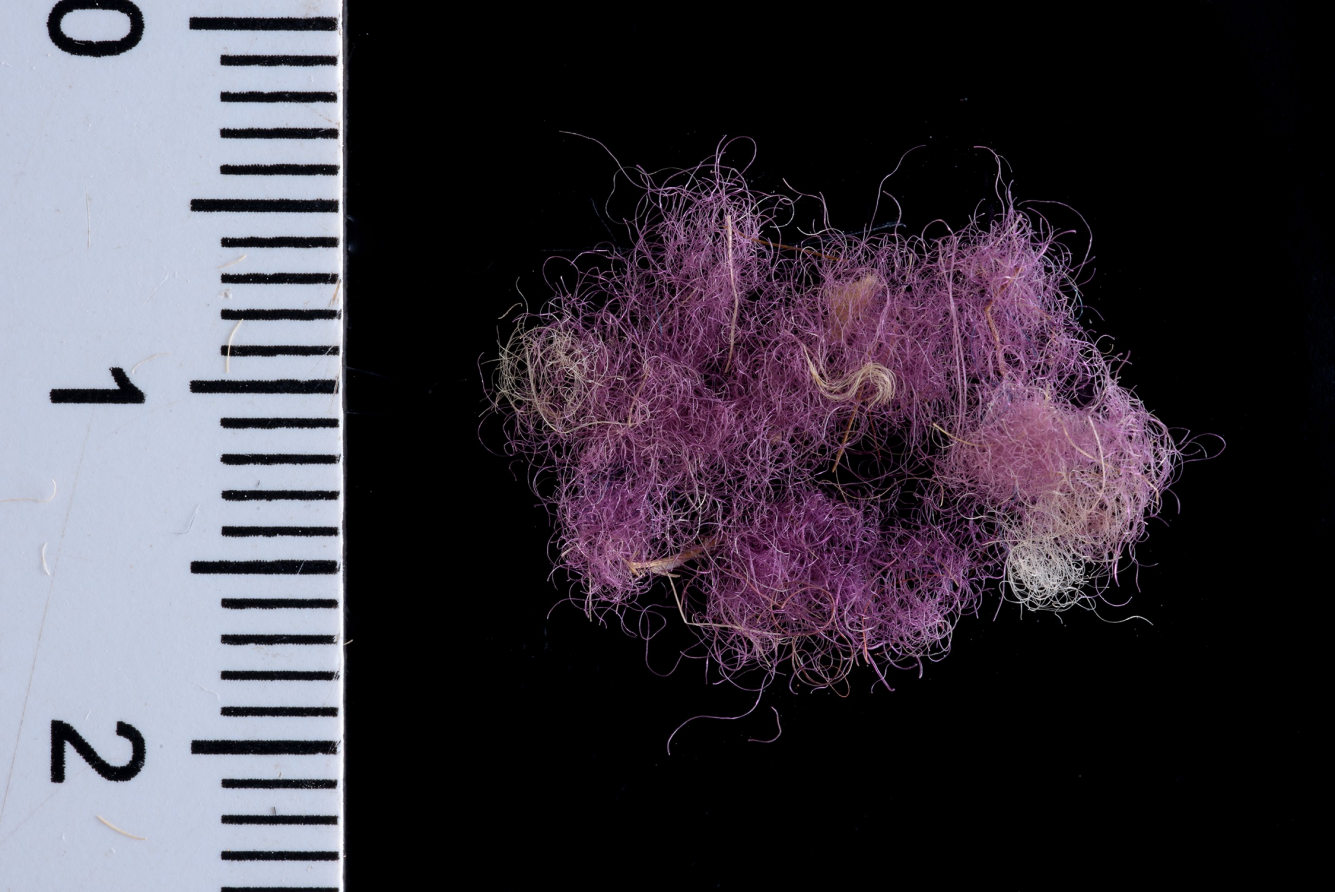
Sample no. 004. Wool fibers dyed pink-purple hue (photo by Dafna Gazit, courtesy of the Israel Antiquities Authority).

**Fig 3:**
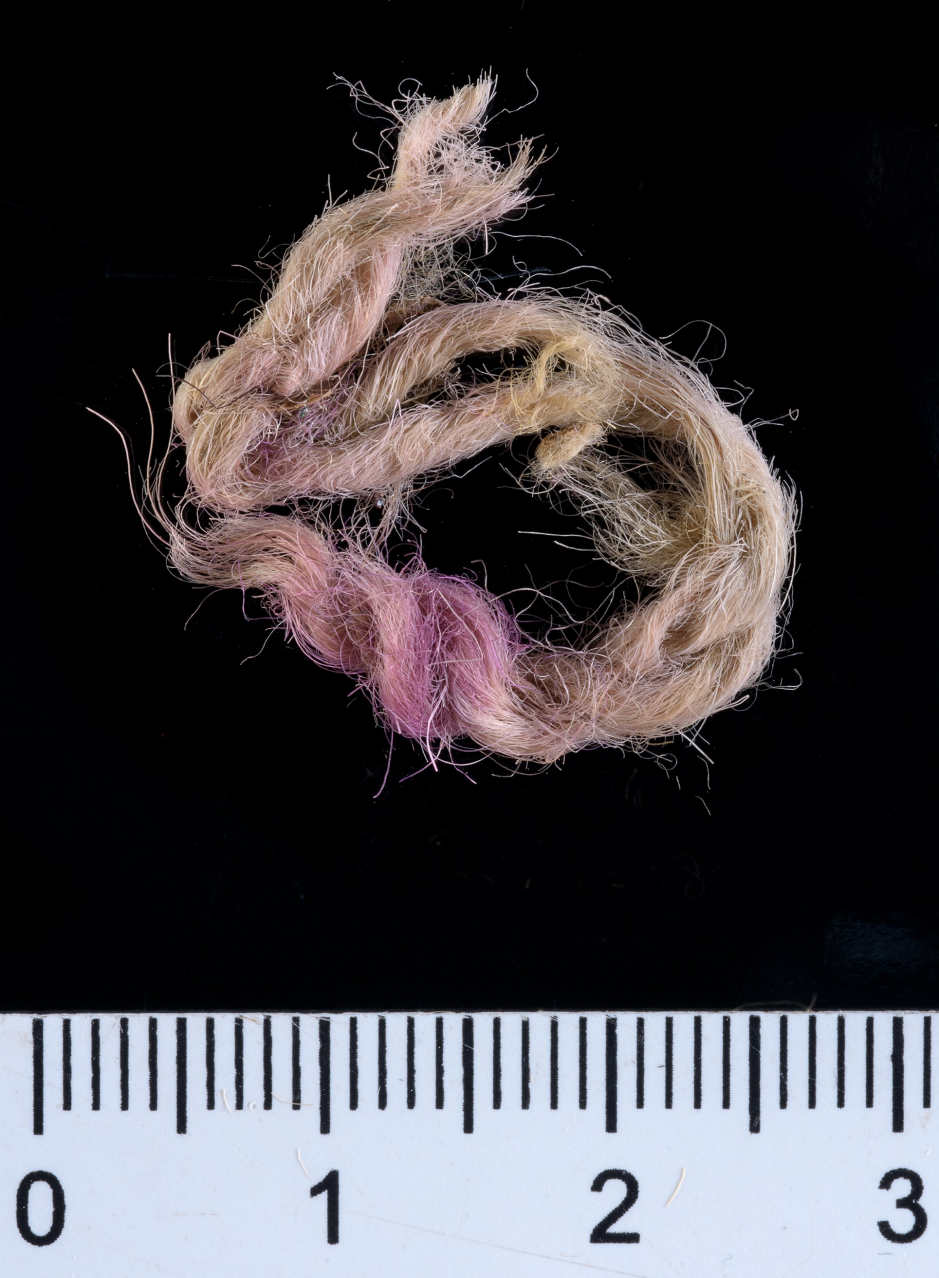
Sample no. 017. A wool string made of two strands plied in Z-direction, with a pink-purple edge (photo by Dafna Gazit, courtesy of the Israel Antiquities Authority).

**Fig 4:**
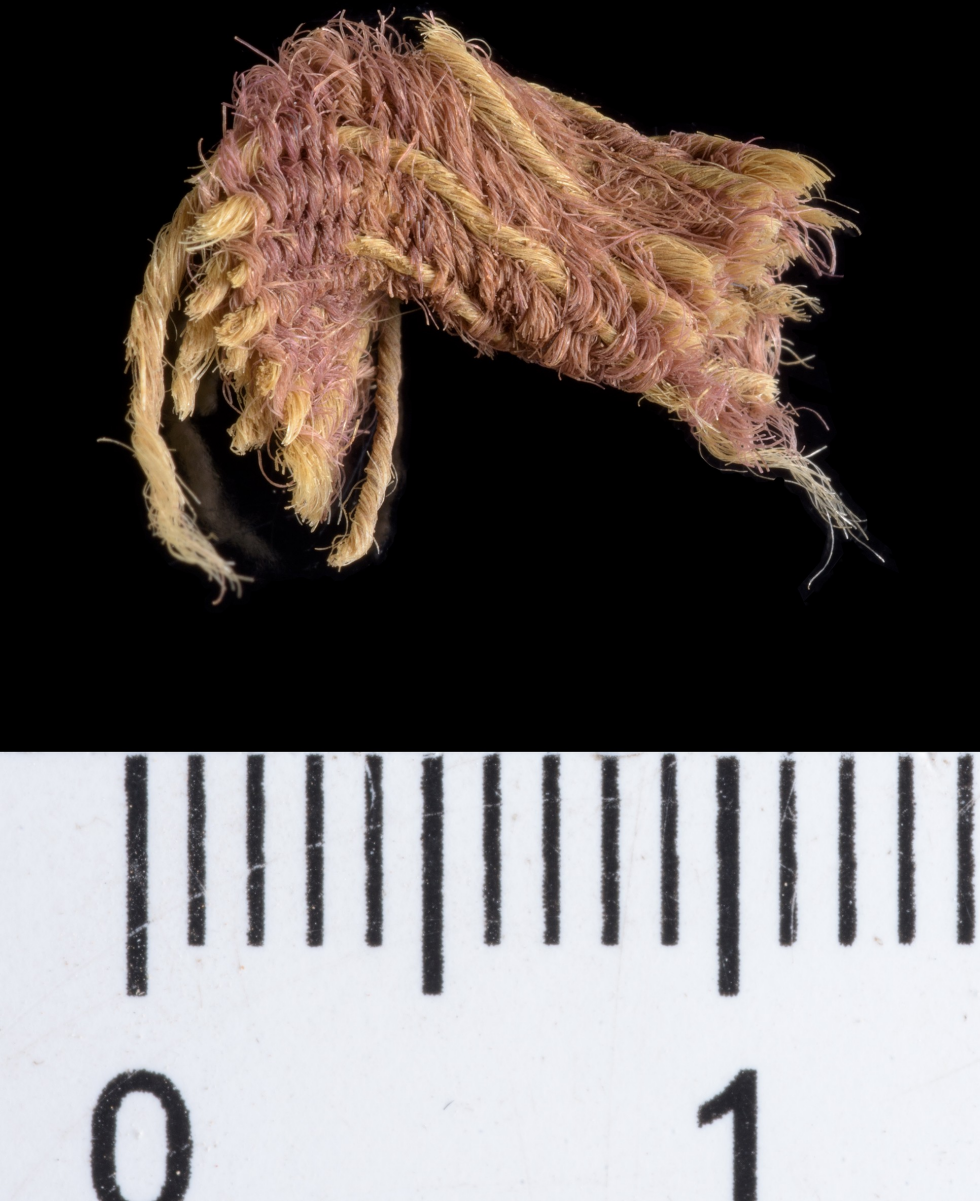
Sample no 018. A wool textile fragment decorated with pink-purple weft threads (photo by Dafna Gazit, courtesy of the Israel Antiquities Authority).

Three of the many fragments of dyed textiles and fibers from Timna that were analyzed, yielded results compatible with true purple dyestuff (nos. 004, 017, 018; see Figs 2–4). They were found in the excavations of Area 13, Site 34 (“Slaves’ Hill”)—one of the largest copper smelting camps in the region [64]. The finds came from a context close to bedrock, defined as a “mixed” locus that represents a shallow deposition of waste embedded in reddish sediments and crushed sandstone (Locus 515, Basket 5644).

The site was tightly dated to the late 11^th^–first half of the 10^th^ century BCE (early Iron Age) based on the results of excavations in several areas. These include a dozen of radiocarbon dates from short-lived materials (mostly date seeds [64]), the study of pottery typology [65] and other considerations [66]. In addition, as part of the current study we sent a fragment from one of the dyed items (no. 017) to be directly dated by radiocarbon. The result (Table 1) is in perfect agreement with the previously published chronology of the site.

**Table 1:** Radiocarbon date for sample no. 017*.

Laboratory number	Locus/basket	Material	Age BP	Cal. BCE
Beta-562244	515/5644	Wool	2850 ± 30 BP	1114–924 cal. BCE (2σ)
				1053–933 cal. BCE (1σ)

* Dating was done at the Beta Analytic Radiocarbon Dating Laboratory; calibration based on IntCal20 atmospheric curve.

**Fig 5:**
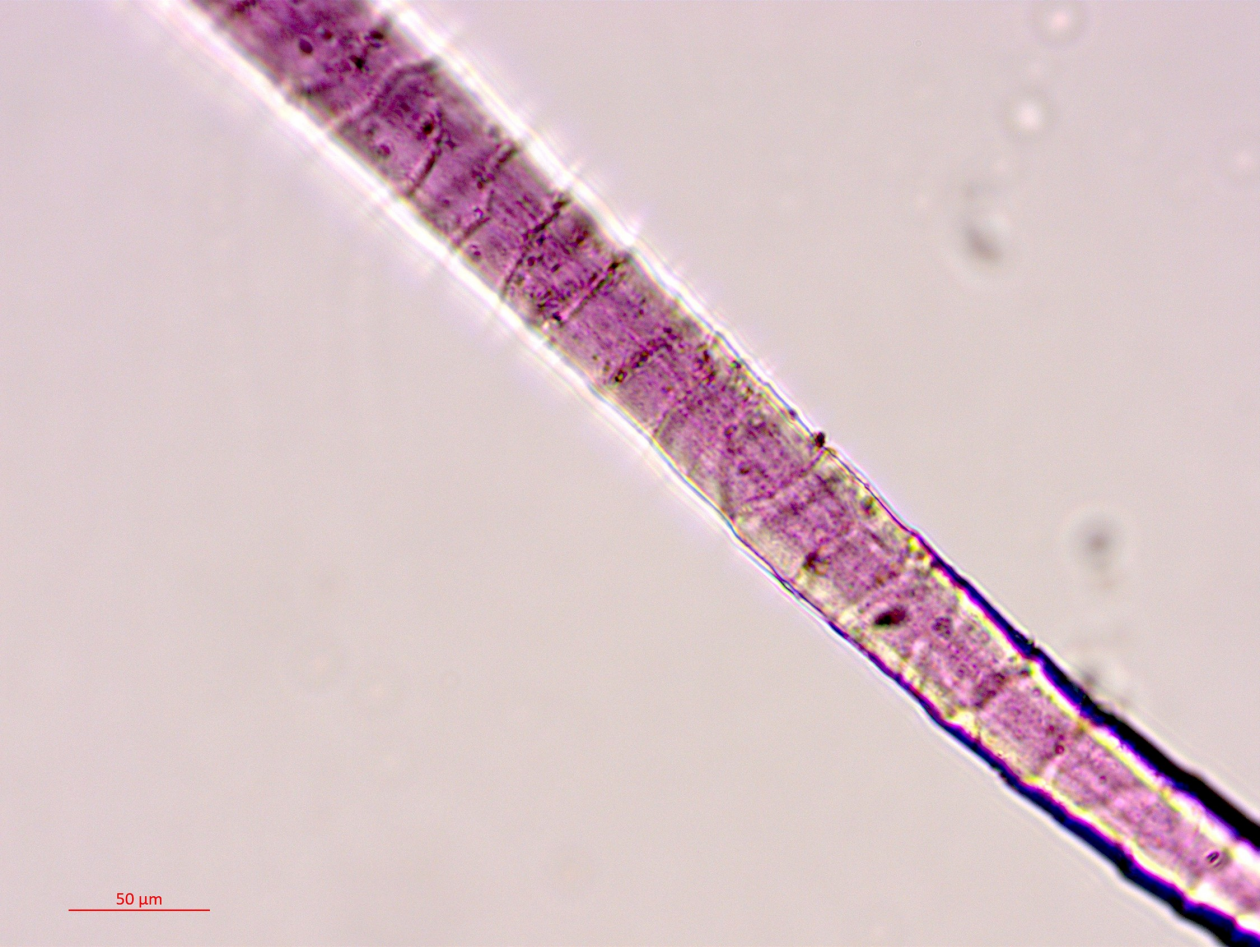
A single fiber of sample no. 004 under a polarizing microscope (photo by Naama Sukenik).

Sample no. 004 was selected from a small group of pink-purplish wool fibers (Fig 2), which were probably ripped from a textile that did not survive. A microscopic examination indicated that the fibers were dyed before they were spun into threads, a standard procedure in the dyeing of wool [10,67]. Sample no. 017 was selected from two threads with a Z-ply (s2Z), 63.8 mm long and 4.9 mm wide, with a pink-purple edge (Fig 3). It is difficult to know the specific function of this string but we can assume that it does not come from a rough textile, but from a decorative fringe or a tassel, both common in the Timna textiles assemblage [68,69] and in the ancient world in general [70,71]. Sample no. 018 (Fig 4) was taken from a textile fragment (1.2 X 0.4 cm). The textile shows a weft-faced tabby weave with uncolored warp threads and decorative pink-purple weft threads. The threads are S-spun. We can assume that this fragment is part of decorative textile bands that are common in the Timna textiles [69]. It is difficult to say with certainty whether all the samples are related to the same textile, but this should not be ruled out (especially samples nos. 004 and 018 that gave similar chemical results–see later). The fiber was identified as wool on the basis of the morphology of its internal layer as observed under a polarizing microscope (Zeiss Axioscope 5) at x500 magnification. Wool fibers (sheep in this case) are characterized by a unique cellular appearance of overlapping scale-like structure (Fig 5), which is unlike any other fiber [72,73], including linen (also found in the Timna assemblage together with goat hair [6]). Wool played an important role in the dyeing industry and was the best raw material for dyeing in the Levant prior to the introduction of cotton and silk [67,74–78]. This is because the protein composition of the fiber allows better absorption of the dye than that of the linen fiber [79,80]. The coarse goat hair on the other hand, was not used for delicate or embellished textiles.

## Methods

Although the composition of the murex dye is not completely clear [27], it is obvious that the final shade is affected by certain colorant components (chromatogram), which can be identified by HPLC analysis in a single measurement: indigotin (IND) and indirubin (INR). The two are found in plant sources such as woad (*Isatis tinctoria L*.) and the indigo plant (*Indigofera tinctoria L*), as well as in several species of shellfish [81–83]. Other components that confer the purple color are present only in mollusk dyes: 6-monobromoindigotin (MBI), monobromoindirubin (MBIR), 6,6-dibromoindigotin (DBI), and 6,6- dibromoindirubin (DBIR), which are the ones that confer the purple color [62,68,83,84]. Other minor components, such as isatinoids and indirubinoids, were also recognized [82], but since they are often absent from archeological textiles, will not be discussed here.

Identifying the organic dye colorants in archaeological textiles is a complex task due to the low concentration of the molecules in the fibers and the limited amount of material available for destructive analysis. In this study we used HPLC analysis, which is considered the most appropriate and reliable method for identifying dyes in archaeological textiles since 1985 [80,85–89]. This method, which is based on chromatography, is efficient in separating and identifying a mixture of compounds and is commonly used in analytical chemistry and biochemistry. It relies on pumps to pass a pressurized liquid solvent containing the sample mixture through a column filled with a solid adsorbent material. Each component in the sample interacts slightly differently with the adsorbent material, causing different flow rates for the different components, and leading to the separation of the components as they flow out of the column, then to be detected by a UV-Visible absorbance detector that quantifies the components in the solution [80,90]. Although the analysis is destructive, it can identify components that are present in minute amounts, and thus only very little quantities of the tested substance are required, ensuring minimal damage with high degree of accuracy and separation capability, qualities that are crucial for the identification of minor components in archeological textiles.

The identification of dyes was based on a database containing known chemical components and wool that was dyed with known dyestuffs and analyzed by the authors prior to the current Timna research [68], and on a series of analyses that were done by the authors in a previous study of wool that was dyed with each of the murex species (*Hexaplex trunculus*, *Bolinus brandaris* and *Stramonita haemastoma*), and analyzed under the same conditions as the archaeological samples ([58,61]; S1 Appendix)

Extracts of dyed textiles were analyzed by the HPLC-DAD (High-Performance Liquid Chromatography with Diode Array Detector) system (Hitachi LaChrom Elite Chromatography), at the HPLC Unit of the Mina and Everard Goodman Faculty of Life Sciences at Bar-Ilan University. HPLC system running EZ Chrom Elite v. 3.2.1 software consisted of an L-2130 binary pump, an L-2200 autosampler, an L-2300 column oven (column temperature of 30°C was used for all analyses), and an L-2455 Diode Array Detector, set to obtain chromatogram spectra in the range of 200-700nm, with extracted chromatograms at 254nm, 454nm, and 554nm. The chromatographic column was a GraceSmart RP18, 5 μm, 250mm × 4.6mm ID. In each test, a characteristic chromatogram was obtained, and the color components were identified by the particulars of their retention time (R_t_) and their characteristic absorbance spectra, including the wavelengths of the absorbance peak in the UV-visible spectrum (λmax).

### Extraction method

The dyes were extracted from the fibers with DMSO (Dimethyl sulfoxide) solvent, which was considered appropriate for indigoid dyes [35,91]. Samples weighing 3 mg each were placed in an Eppendorf test tube (1.5 mL), along with 150 μl of DMSO. Each sample was heated to 95°C in a water bath for 10 minutes. By that time, the solution turned blue, and was then separated from the settled sediment and transferred to a clean Eppendorf test-tube. Finally, the sample was subjected to centrifugation for 5 minutes at 12,000 g., and 20 μl of the supernatant was injected into the HPLC system’ HPLC.

### HPLC analysis protocol

The mobile phase for the protocol that was used in this study was made up of A: phosphoric acid 0.5% (w/v); B: methanol; and C: H_2_O. The flow rate was held at 1 ml/min, and the injection size was 25 μL. Gradient elution conditions are tabulated in Table 2.

**Table 2:** Linear gradient elution for HPLC-DAD analysis of the extracted purple dyes.

Time (min)	% A	% B	% C
0	10	50	40
3	10	80	10
20	10	80	10
25	10	90	0
30	10	90	0
33	10	50	40
40	10	50	40

## Results

**Fig 6:**
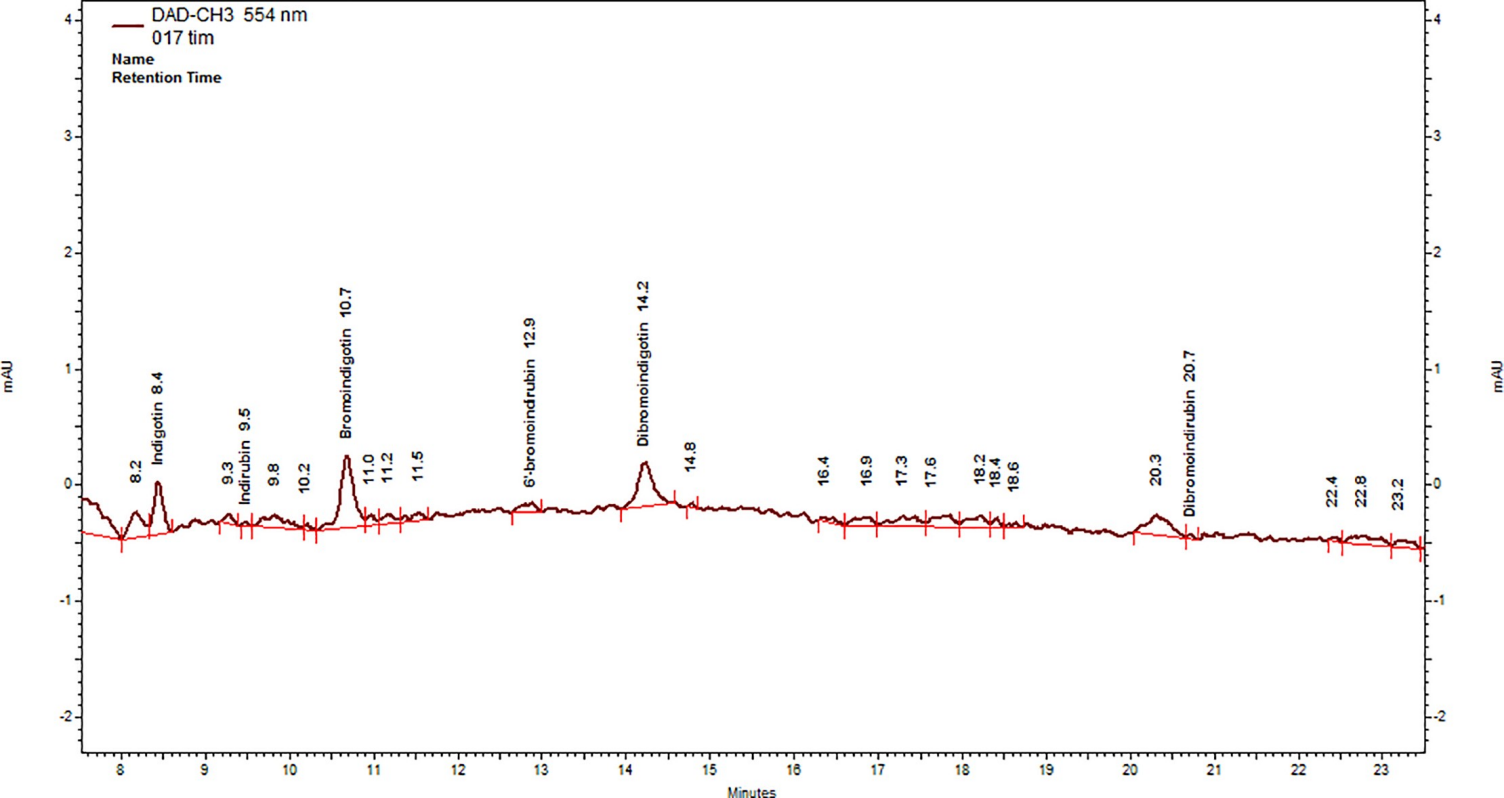
HPLC chromatogram of sample 017. Extracted using DMSO, wavelength 554 nm.

According to the analysis result at 554 nm (Table 3), the archaeological samples were dyed with true purple. They contain two molecules that are principal markers of this color, namely MBI and DBI. The chromatogram of sample no. 004 shows only two pronounced peaks at 554 nm: MBI was detected at 10.75 R_t_ with a typical spectrum (242nm, 288 nm, 347, 608nm λ_max_), and main peak at 14.40 R_t_ of DBI with a typical spectrum (292 nm, 307nm, 353 nm, 596nm λ_max_). The other components did not appear in a significant amount. The chromatogram of sample no. 017 has a few peaks at 554 nm (Fig 6): IND was detected at 8.46 R_t_ and with a typical spectrum (242nm, 286 nm, 339 nm, 613nm λ_max_), MBI was detected at 10.68 R_t_ (242 nm, 289 nm, 346 nm 608 nm λ_max_), DBI at 14.23 R_t_ with a typical spectrum (292 nm, 304nm, 350 nm, 603nm λ_max_) and DBIR was detected at 20.3 R_t_ with typical spectrum (304nm, 368nm, 553 nm λ_max_). The chromatogram of sample no. 018 similar to sample 004 shows main peak of DBI at 14.40 R_t_ with a typical spectrum (291 nm, 302nm, 353 nm, 601nm λ_max_) and minor peak at 10.7 of MBI (240nm, 347nm 610nm λ_max_). It can therefore be determined with certainty that the fibers in all three samples were dyed with genuine shellfish dye.

**Table 3:** Analytical results, including retention time, spectral wavelengths identification and the relative percentage of the components that were identified at 554 nm.

IAA Number		IND	MBI	DBI	DBIR
004	Retention time (minutes) (λmax nm)	8.5	10.7 (242, 288, 347, 608)	R_t_ 14.4 (292, 307, 353, 596)	20.6
	Relative percentages % of the component	1%<	14%	86%	1%<
017	Retention time (minutes) (λmax nm)	R_t_ 8.45 (242, 286, 339, 613)	R_t_ 10.68 (242, 289, 346, 608)	R_t_14.2 (292, 304, 350, 603)	R_t_ 20.3 (304, 368 553)
	Relative percentages % of the component	11.10%	15.60%	63.87%	9.40%
018	Retention time (minutes) (λmax nm)		10.7 (240, 347, 610)	14.4 (291, 302, 353, 601	
	Relative percentages % of the component	1%<	14.28%	85.71%	1%<

Distinguishing between the three Mediterranean molluscan species, *H*. *trunculus*, *B*. *brandaris* and *S*. *haemastoma*, is not easy because numerous variables (*e*.*g*. geographical location, age and sex of the snails, the dyeing process, analytical methods employed to record the composition etc.) may affect the reported composition of the purple dye [10,84,92]. Nevertheless, it is possible to see a clear trend with respect to the ratios of the dye substances in each species [27,35,58,61,84,91,93], that helps to identify the species of the murex used in the dyeing of the archaeological textiles with a high level of certainty.

**Fig 7:**
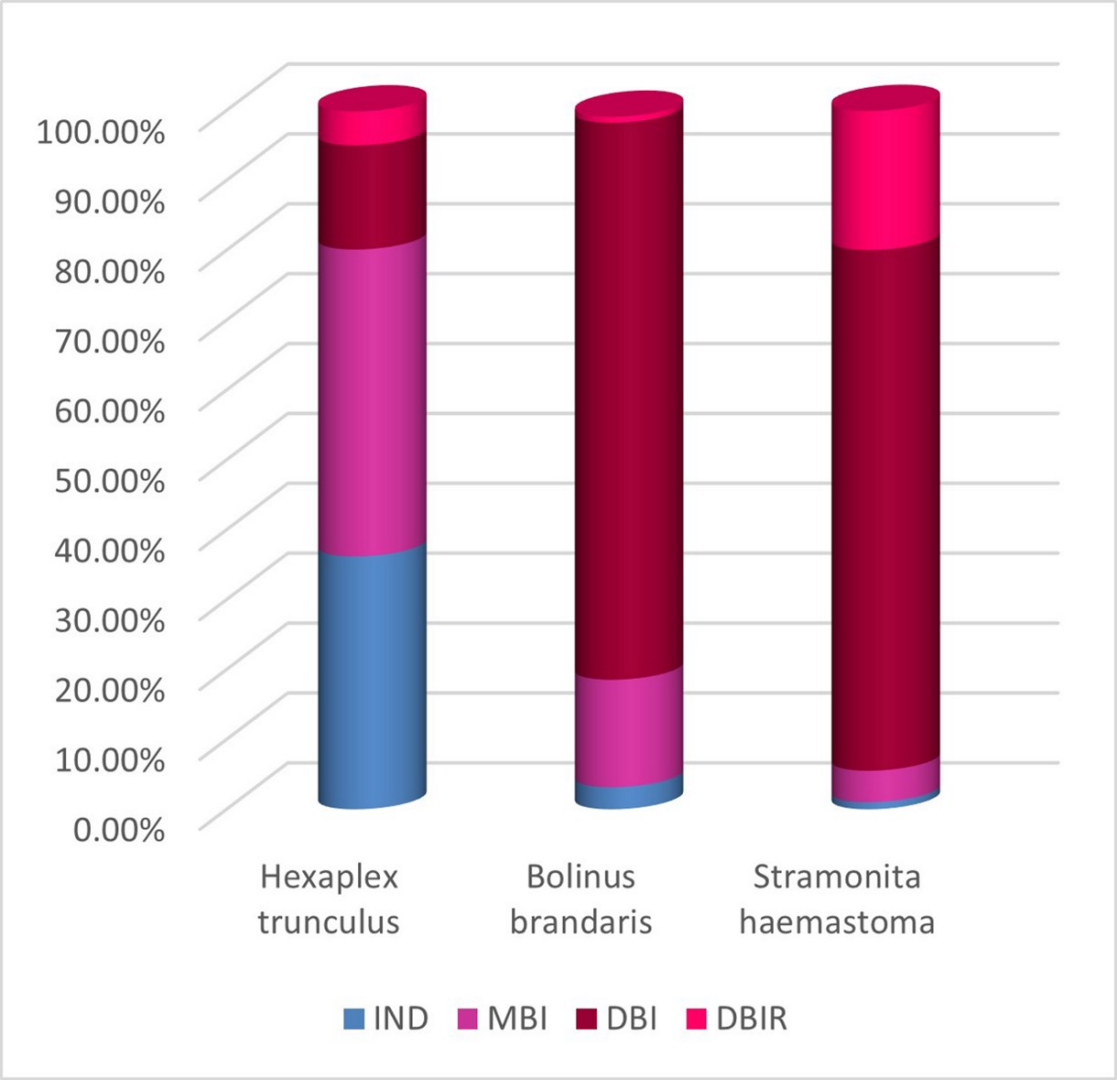
Relative proportions of dye components (IND; MBI; DBI; DBIR) at 554 nm in modern fleeces dyed with the three species of sea mollusks (the graph shows the average results of all the modern samples).

Examination of the percentage of the colorants and the proportions of the components in chromatograms that were done by the authors in pervious analyses (Fig 7 and S1 Appendix; [58,61,94]), which is in agreement with other previous studies (see for example: [35,84,91]), shows that in most cases *H*. *trunculus* contains relatively high concentration of IND (indigotin) and high concentration of MBI (monobromoindigotin) compound, while DBI (*6*,*6-dibromoindigotin*) is present in relatively small quantities; in *B*. *brandaris* and *S*. *haemastoma* on the other hand, DBI compound is the most abundant (over 60%), but the proportion of IND in dyes derived from these two mollusks is low (less than 5%) and it cannot always be detected in the chromatography (Fig 7; S1 Appendix: Table 1; see also [61,84,93,95,96]).

**Fig 8:**
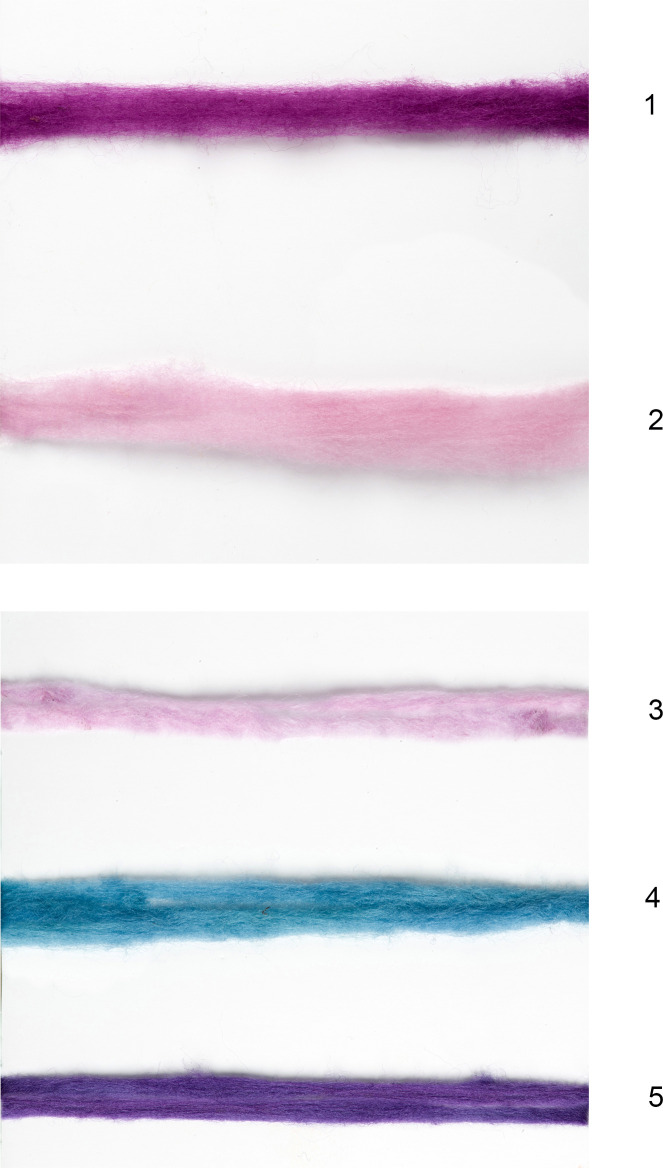
Wool fleeces dyed with different species of sea snails. 1–2: *S*. *haemastoma*; 3. *B*. *brandaris*; 4–5: *H*. *trunculus* (Dyeing—Zohar Amar and Naama Sukenik. Photo by Shahar Cohen).

According to the chromatogram of samples nos. 004 and 018 only two components were detected. The most abundant colorant is DBI (85%-86%), followed by MBI (14%-15%), while no IND and DBIR components were found in significant amounts (Table 3). Hence, it can be determined that samples were colored with a dye substance obtained from *B*. *brandaris* or *S*. *haemastoma*, which are characterized by a relatively high concentration of DBI compound and low concentration of IND (see also [91]). The experimental dyeing with this species strengthens our conclusion, because while *H*. *trunculus* yielded more violet-blue hues due to the indigo compound, the two other species produce a more pink-purple hue (Fig 8; S1 Appendix), due to the high concentration of the combined DBI and the DBIR components which are responsible for the characteristic red/pink hue (Table 3 [97]). Although a separation of *B*. *brandaris* and *S*. *haemastoma* species is not possible [35,91], it should be noted that according to the experimental dyeing results, *S*. *haemastoma* is a little easier to use than *B*. *brandaris* due to the difference in gland-size (an average of 0.6 gram in *S*. *haemastoma*, an average of 0.2 gram in B. *brandaris*), and it produces a hue of reddish-purple similar to that of the archaeological fiber (see S1 Appendix).

According to the chromatogram of sample no. 017, the DBI compound appears in a relatively high concentration (63.87%), which presents the relatively high peak areas measured at 554 nm, followed by MBI (15.60%), IND (11.10%) and DBIR (9.40%) (Table 3, Fig 7). Although all the components that are known in each of the three species of murex were found in the same sample (include DBIR components, see Fig 6), the proportions that are seen in the chromatogram, with high concentration of DBI but still relatively large concentrations (above 8% each) of MBI, IND and DBIR, do not fit any of the typical murex species. This composition could result, according to Koren (e.g., [19,84]), from numerous variables in *H*. *trunculus*, including a type of *H*. *trunculus* that is indigo-poor and DBI- rich. However, in our opinion it is more likely to be the outcome of a double dyeing method using either *B*. *brandaris* or *S*. *haemastoma* in combination with *H*. *trunculus*, a method that was mentioned by Pliny the Elder for obtaining reddish-purple colors (Plinius, NH: IX, 62, 137). A series of dyeing experiments that was conducted by the authors shows that although *B*. *brandaris* or *S*. *haemastoma* produce very beautiful color, the quantity obtained is small. Adding a few glands of *H*. *trunculus* helps in increasing the amount of color that is absorbed into the fibres (S1 Appendix). Doumet [98] already noted the importance of the enzymes (purpurase) existing in *H*. *trunculus* for the dyeing process, and showed that a better result was achieved when 10% of *H*. *trunculus* glands were added to a *B*. *brandaris* or *S*. *haemastoma* bath dye (see also [21,99]). However, it is important to refer to our proposal with caution. More information about the origin of the purple pigments and the impact of the variables, is necessary before a clear conclusion can be drawn in the future.

## Discussion

### Observations on the purple dyeing industry of the early iron age

The dyed textiles from Timna constitute a corpus of paramount importance, as no parallels are known to us today from the Southern Levant. Their analyses enhance our knowledge of garment and textile production technologies in the early Iron Age (~1100–900 BCE). The results of the present study indicate that the pigments present in three different samples of fiber are remnants of true purple dye. While pre-Roman evidence of the use of this dye in the Southern Levant exists in the form of murex shells heaps (*e*.*g*.[52,100] or stained ceramics (*e*.*g*. [19,56,58,84]), until now none of the surviving pre-Roman textile fragments have tested positive for a true purple dye.

According to our results the dyeing process of all samples was based on *B*. *brandaris* or *S*. *haemastoma* (the analytical results did not allow distinguishing between these two species). In one sample (no. 017) *H*. *trunculus* was most probably also used, and this sample therefore represents the double-dyeing process for the production of the expensive dye of Tyrian purple, which was described by Pliny the Elder (NH: IX, 62, 137). According to Pliny, in this method the fiber was dipped into a first bath of ‘*pelagium’* and then into a second bath of ‘*bucinum’*. Although the identification of these species is not certain, it is agreed by all researchers that Pliny refers to two different species of murex [10,101–103]. It is important to emphasize that the two species were added to enhance the reddish color [19], as seems to be the case for the Timna sample (here probably *H*. *trunculus* with *B*. *brandaris* or *S*. *haemastoma*).

**Fig 9:**
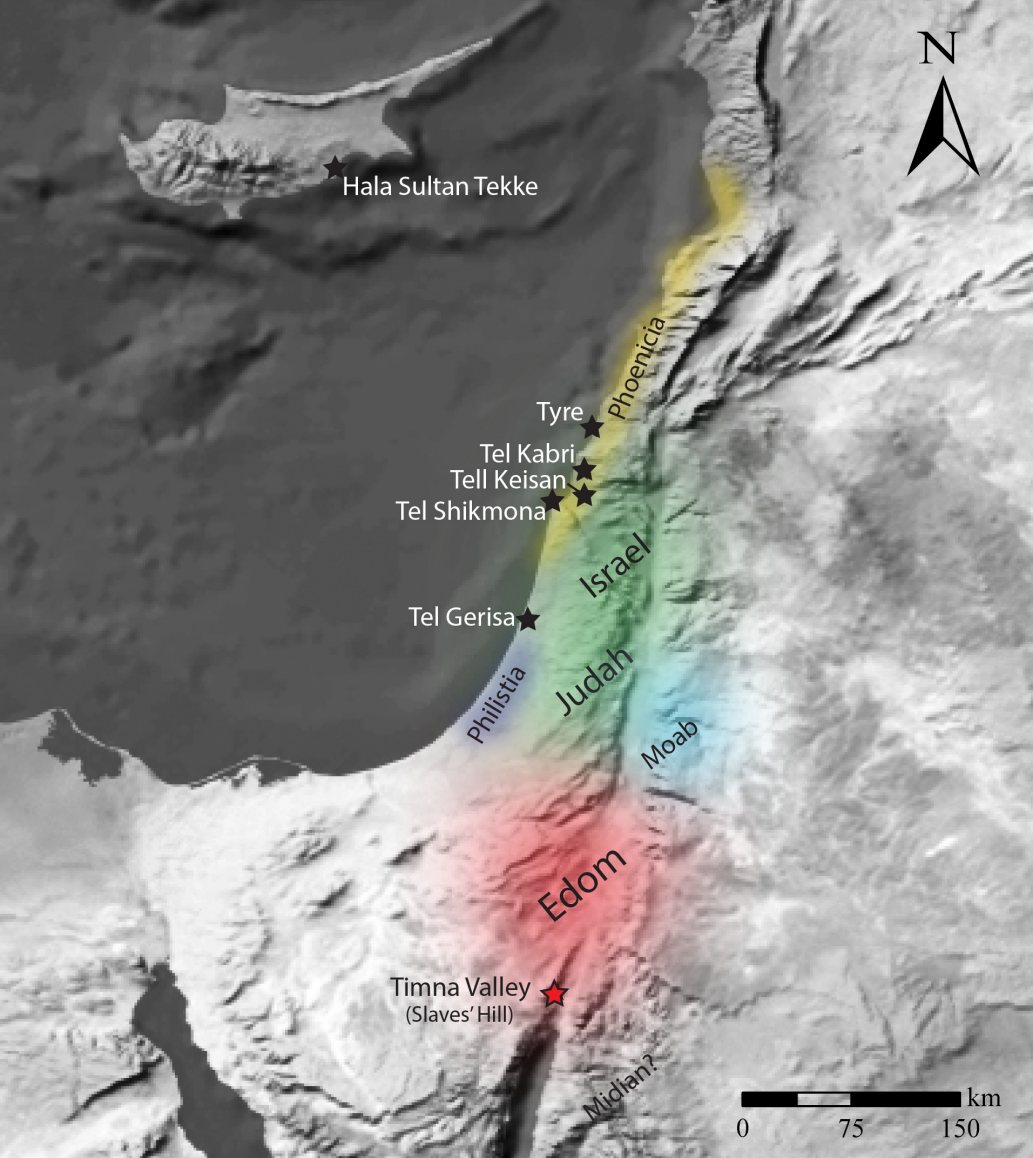
Location of “Slaves’ Hill” (Timna Valley) and other iron age sites with evidence of the true purple industry. (Base map made with Natural Earth, free vector and raster map data @ naturalearthdata.com).

It is reasonable to suggest that the Timna samples are a product of the dyeing industry in the Phoenician Coastal Plain. The three murex species discussed above are present in the Mediterranean Sea and are not found in the Red Sea [104,105]. In the Eastern Mediterranean, the largest concentration of Iron Age sites with evidence of purple industry is located along the northern coast of the Southern Levant [15] (Fig 9). They include Tyre, Shikmona, Tel Kabri and Tel Keisen [16,52,56,58,100,106]. The only other sites with possible evidence of Iron Age purple industry currently known in the wider Eastern Mediterranean are Hala Sultan Tekke in Cyprus [52] and Tel Garisa in modern day Israel [16].

Another species of murex—*Virgin murex* (*Chicoreus virgineus*, also from the family *Muricidae*)—is found in the Red Sea [107]. Although there is no direct evidence of dyeing with this species, a few of its shells were reported from the Roman sites of Myos Hormos (Egypt) and the port of Hafun (Somalia), raising the possibility that it was used for dyeing [108]. However, the scarcity of the finds, coupled with the absence of historical evidence makes the possibility of the use of this snail in the production of the Timna samples much less likely.

It is important to emphasize that in Egypt, only one site related to the purple industry has been found so far; it is dated to the Late Bronze and the Roman periods (Marsa Matruh, [52]). Additionally, the identification of mollusk purple in Egyptian textiles is rare prior to the Roman Period [52,109–111]. For example, in the Amarna textiles no indication of true purple was found [112], and the same is the case for other assemblages of high quality textiles (related to the pharaohs and high officials) from Middle Kingdom and New Kingdom sites [113]. The exclusion of Egypt as the source of the purple dyed textiles of Timna is in accord with our assumption that all wool textiles of this assemblage were not obtained from Egypt, which specialized in growing flax and the production of linen [68,69]

### Observations on the early iron age society of Timna Valley

The find of fibers dyed with true purple in Timna adds to a growing body of evidence for the complexity of the society operating the copper mines during the early Iron Age [64,68,69]. This period, which followed the collapse Egypt and the end of its control over the Southern Levant [114], is typically considered an era of limited inter-regional connections, although this is starting to change with the advance of the application of archaeological sciences [115]. The societies are assumed to be quite fragmented, and the timing of the emergence of local polities of historical significance, such as the kingdoms of ancient Israel, Edom and Moab, is hotly debated, as it has implications on our understanding of the historicity of the Old Testament (e.g. [116]).

The identification of the early Iron Age society of Timna Valley and the nearby regions with biblical Edom is discussed elsewhere ([117] and references therein)]. The discovery of true purple-dyed textiles renders further support to the suggestion that this society was part of a *kingdom* (the Edomite Kingdom) already in this period [117–119], as it provides strong evidence for the presence of elite (see also a study based on diet [120], and direct evidence for long-distance trade connections (see also [68,118]). There is little doubt that the expensive true-purple garments were used as a marker for social status, and had a role in establishing a hierarchical social structure in the fledgling kingdom. In that regard, it is interesting to note the biblical description of the *kings* of Midian–a neighboring kingdom to Edom (although without archaeological identification)–who wore “purple [ארגמן] garments” (Judges 8:26). This reference is traditionally dated to the period under discussion, in which tribal nomadic societies consolidated into strong polities, typically without leaving any conspicuous archaeological remains [121,122]. The uniqueness of the case of Edom is twofold, as it involves both an exceptionally rich archaeological record of nomads (the result of mining and smelting activities) and an unprecedented preservation of organic materials (the result of the extreme arid climate). The latter provides a window into various aspects of life of the early Edomites, and–by proxy—of other peoples and cultures of the broader region in this period.

The true-purple dyed textiles were found within industrial waste, in association with smelting activities. This further stresses previous observations on the important role of metalworkers within this society; the smelters, holding knowledge of one of the most sophisticated crafts of the time, were part of—or in close association with—the elite of the society, with access to excellent and exotic foods [120,123], and luxurious commodities, including those represented by the new finds. The new evidence also provides insights into elite “fashion”—in early Iron Age Timna and probably beyond. In addition to the decorations in red and blue dyes from plant sources [68], we can now conclude the use of decorations with the threads of the prestigious true purple. Colored clothing was the preference of elite in the Ancient Near Eastern societies [70]; yet, we know very little about the variety of decoration choices. In this study, sample no. 018, a decorated textile, and sample no. 017 that seems to be an element of a fringe or tassel, were probably part of a garment of a prestigious wardrobe [69,70]. It is interesting to note that in all known cases, the archaeological textiles with evidence of purple (from the Roman period) were not completely dyed with this expensive color, but incorporated purple threads as only one element of the textile weaving (usually in the *weft*), and sometimes only in un-woven decorative elements [124]. In most cases these textiles are very delicate and represent a high quality of weaving [15]. The correlation between high-quality textiles and expensive dyestuff is in accord with the textual evidence (see details in the Introduction above).

To conclude, this is the first time that a 3000 years old wool textile was found in the Southern Levant with evidence of shellfish-based dyes. This find helps researchers to reconstruct the complete *chaîne opératoire* of the true purple industry of the Iron Age, starting with the archaeological evidence of shell heaps in various sites along the Mediterranean coast, through finds of the dyeing vessels (stained ceramic basins), to the final product of textiles decorated with the most expensive dyestuff in the ancient world. Only three fragments survived, but they open a window into a wide range of social aspects, from the lives of Iron Age elite, to the means of establishing social status in the kingdoms of the region, including those based on nomads [121].

## Supporting information

S1 Appendix (DOCX)
