# Supplementary material for: Early evidence of royal purple dyed textile from Timna Valley (Israel)
Source: PLoS One. 2021 Jan 28;16(1):e0245897. doi: 10.1371/journal.pone.0245897 (PMC7842898; doi:10.1371/journal.pone.0245897)
Supplement: S1 Appendix — (DOCX) [file pone.0245897.s001.docx]

**Appendix A: Experimental Purple Dyeing**

Between 2009 and 2012, a series of experiments was conducted in the lab to reconstruct the dyeing process using the hydrosulfite vat method on three different species of murex: *Hexaplex trunculus*, *Bolinus brandaris* and *Stramonita haemastoma* (see also [1]) .

## *Obtaining snails*

The snails were collected from three different sources:

1. From Italy: The *H.* *trunculus*, and *B. brandaris* snails, were bought alive from the fishermen from the port of Venice and *S. haemastoma* were bought alive from Palermo, Sicily. The snails were then cracked, and the glands were removes and dried by Prof. Zohar Amar in the lab at Milan, Italy [2] and few were brought to Israel for dyeing.

2. Snails which were collected in Israel near Haifa (with special approval from the Israel Nature and Parks Authority).

3. A few dry glands were received from Petil Tekhelet, a non-profit organization which bought the snails from Croatia. The dyeing process was done in the lab in Bar Ilan University.

***The dyeing processes***

Three grams of sodium hydroxide were added to 0.2-3 grams of crushed dyestuff in 200 ml tap water (at 80° Celsius) and the solution was stirred. Twelve grams of sodium hydrosulfite (Na_2_S_2_O_4_) were then mixed in and stirred slowly to avoid oxidation, followed by 4.5 grams of household-grade citric acid. The wool sample was then placed in a beaker with the dyeing solution and incubated for at least 15 minutes before it was removed and exposed to air (Fig A1).

Fig A1: A diagram of the dyeing experiment

A wide range of variables were tested during the various experiments, including: degree of exposures to light; dyeing with seawater or sweet water; different of alkaline material and double dyeing with two different species, etc. [1]. The main conclusions that emerged from the experiments are as follows:

- There is significant difference in the hue obtained by *Hexaplex trunculus* which gives more violet-blue hues and from *Bolinus brandaris* and *Stramonita haemastoma* that give more reddish-purple hue.
- All species are very sensitive to light and give more blue shades under increased sunlight exposure. The dyeing with *Hexaplex trunculus* gives the best blue shade (similar blue hue were received with dyeing in seawater or by heating the wool fleece after dyeing).
- We do not find a significant change in the dyeing results between snails that were imported from Italy, Croatia or from those from Israel. At the same time, any slight change in the dyeing process itself causes a different result.
- In the hydrosulfite vat method, we do not find a significant difference between dyeing with dry or fresh glands.
- Various alkaline materials can be used to produce an alkaline environment in the vat dyeing, include natural natron, wood ash, urine, broken shells etc.
- *H. trunculus* yields a much higher quantity of dye than the two other species due to the difference in the gland size (see also [3]).

- Dyeing with *S. haemastoma* is a little easier than dyeing with *B. brandaris* due to the difference in gland size and the fact that dyeing with the latter results in a weaker color.
- Double dyeing with *B. brandaris* and *H. trunculus* enriched the dyeing results.

***Analytical study and results***

From the experimentally-dyed materials, fourteen wool samples were analyzed by HPLC-DAD method: four were dyed with *Hexaplex trunculus*, three with *Bolinus brandaris*, and seven with *Stramonita haemastoma*. The samples were extracted and analyzed under the same conditions as the archaeological samples (described in detail under the Methods section in the main article). Several components were identified according to their retention time (Rt) and the absorbance spectra in the UV-visible spectrum (λmax), but we focus on the dominant components: indigotin (IND) and its derivatives, monobromoindigotin (MBI) and dibromoindigotin (DBI), with absorbance maxima between 601 nm and 613 nm; and dibromoindirubin (DBIR) with absorbance maxima between 536 nm and 544 nm. The percentage of the colorants and the proportions of the components in chromatograms are shown in Table A1. The experimental data [published previously in 4–6], are used in the current study to interpret the results from the ancient samples.

| DBIR | DBI | MBI | IND | Murex species |
| --- | --- | --- | --- | --- |
| 2.28 | 7.8 | 40.83 | 50.00 | *H. trunculus* |
| 6.67 | 8.54 | 49.82 | 34.916 | *H. trunculus* |
| 10.42 | 20.82 | 46.12 | 22.01 | *H. trunculus* |
| 0 | 23.25 | 38.92 | 37.81 | *H. trunculus* |
| 0 | 82.09 | 13.41 | 4.47 | *H. branaris* |
| 1.52 | 71.95 | 22.59 | 3.94 | *H. branaris* |
| 1 | 85.11 | 10.12 | 1.07 | *H. branaris* |
| 19.89 | 74.5 | 4.5 | 1.022 | *P. haemastoma* |
| 26.68 | 68.84 | 4.3 | 0 | *P. haemastoma* |
| 5.23 | 89 | 4.26 | 1.41 | *P. haemastoma* |
| 16.51 | 69.01 | 9.87 | 4.59 | *P. haemastoma* |
| 7.1 | 91.13 | 1.76 | 0 | *P. haemastoma* |
| 25.48 | 68.6 | 5.85 | 0 | *P. haemastoma* |
| 31.51 | 62.99 | 4.31 | 1.16 | *P. haemastoma* |

Table A1: Relative percent (%) of dye components (IND/MBI/DBI/DBIR) in the modern samples that dyed with mollusk species (calculated at 554nm)

Bibliography

1. Amar Z. The Argaman (Purple): Porphura and Arjawan in Jewish Sources and Further Inquiries into Tekhelet. Har Bracha. Har Bracha; 2014. (Hebrew)

2. Amar Z. A practical Guide to Producing Raw Dye from Snails for Royal Purple and Biblical Blue : History and Reality. Neve Tzuf; 2020. doi:https://www.zoharamar.org.il/wp-content/uploads/Short-Guide-to-Cracking-the-MurexPDF.pdf

3. Cardon D. Natural Dyes, Sources, Tradition, Technology and Science. London: Archetype; 2007.

4. Sukenik N. Dyes in Textiles from the Early Roman Period Found in the Judean Desert Caves: Chemical, Historical and Archaeological Aspects. PHd, Thesis. Bar-Ilan University (Hebrew). 2013.

5. Sukenik N, Iluz D, Amar Z, Varvak A, Bar S. New Evidence of the Purple-Dye Industry at Tel Shiqmona, Israel. Arcaeometry. 2017;59: 775–785. doi:10.1111/arcm.12290

6. Sukenik N, Varvak A, Amar Z, Iluz D. Chemical Analysis of Murex-Dyed Textiles from Wadi Murabba’at, Israel. J Archaeol Sci Reports. 2015;3: 565–570. doi:10.1016/j.jasrep.2015.08.003
